# Comparison of Four Diagnostic Methods for Detection and Relative Quantification of *Haemonchus contortus* Eggs in Feces Samples

**DOI:** 10.3389/fvets.2017.00239

**Published:** 2018-01-24

**Authors:** Sara Ljungström, Lynsey Melville, Philip John Skuce, Johan Höglund

**Affiliations:** ^1^Department of Biomedical Sciences and Veterinary Public Health, Section for Parasitology, Swedish University of Agricultural Sciences, Uppsala, Sweden; ^2^Moredun Research Institute, Edinburgh, United Kingdom

**Keywords:** coproscopy, fecal egg counts, McMaster, peanut lectin, DNA methods, loop-mediated isothermal amplification, real-time polymerase chain reaction, ruminants

## Abstract

We compared four methods for identification of *Haemonchus contortus* eggs. With increased trade in animals within and between countries and continents, it has become important to correctly identify *H. contortus* eggs in fecal samples. To validate the outcome of diagnostic tests, sheep feces (*n* = 38) were collected from naturally infected flocks in Sweden. Subsamples were analyzed with (a) McMaster egg counting; (b) differential counting of eggs after staining with peanut agglutinin (PNA); (c) detection of DNA following amplification by real-time quantitative polymerase chain reaction (qPCR); and (d) loop-mediated isothermal amplification (LAMP). Differences between similar tests (microscopic and molecular) and SD (±SD) were analyzed with Bland–Altman plots and Spearman rank correlation. Strongylid egg counts ranged from 200 to 12,100 eggs per gram (epg) (mean epg ± SD = 1,278 ± 2,049). Microscopy showed presence of *H. contortus* eggs in 27 (73%) unstained samples and in 28 (76%) samples stained with PNA, whereas 29 samples (78%) tested positive in LAMP and 34 (91%) in qPCR analysis. The cycle threshold (Ct) values with LAMP ranged between 13 and 38 (mean ± SD = 21 ± 7), and those in qPCR between 25 and 49 (mean ± SD = 33 ± 6). In the LAMP and qPCR analyses, seven (19%) and three (8%) samples, respectively, had a cycle threshold (Ct) >35, whereas no reactions were observed in eight (22%) and three (8%) samples, respectively. There was good agreement between the diagnostic tests based on microscopic examination and DNA detection, although the molecular tests were more sensitive. The bias between the microscopy methods (−4.2 ± 11) was smaller than for the molecular tests (−9.8 ± 10). The observed ranking in terms of test sensitivity was: McMaster counting by conventional microscopy < PNA < LAMP < qPCR. In conclusion, *H. contortus* can be identified by McMaster counting, without major mistakes regarding false positive results. However, molecular methods provide the capacity to diagnose *H. contortus* eggs with increased accuracy. This is essential when animals are investigated in quarantine or in studies evaluating anthelmintic treatment efficacy. These methods could also be applied to fecal samples from wildlife to investigate nematode transmission between wildlife and livestock.

## Introduction

*Haemonchus contortus* is a cosmopolitan blood-feeding parasite in the abomasum of ruminants. It is recognized as one of the greatest health problems in small ruminants, leading to reduced production and loss of income for farmers throughout the world (1). Recent studies of small ruminants have identified the presence of multidrug resistant *H. contortus* in Sweden (2). This makes correct diagnosis of this damaging parasite in feces samples more critical than ever. In routine testing for gastrointestinal nematodes (GIN), it is thus naturally of the ultimate importance to have correct information about the species involved and especially whether *H. contortus* is present. For example, introduction of ivermectin resistant *H. contortus* along with imports of sheep to Sweden from mainland Europe has been reported (2). It has also been demonstrated that *H. contortus* survived fenbendazole treatment in European bison (*Bison bonasus*) used for restocking purposes (3). Furthermore, parasitological screening of moose (*Alces alces*) demonstrated low levels of *Haemonchus* (4). Parasitological screening of the abomasal contents have also been used to detect *H. contortus*, in samples from wild deer (5), and helped demonstrate the transmission of anthelmintic-resistant parasites between livestock and wildlife.

The presence of *H. contortus* in feces can be detected either by microscopic identification of eggs or ideally from cultured larvae (6, 7). To date, these diagnostic methods have been widely used to detect *H. contortus* infection in both wild and domestic ruminants. Although microscopy can be considered a cheap and fairly reliable method, morphological identification of parasite stages in feces samples requires expertise in the form of trained technical staff. In the long run, this makes the method impractical in a routine diagnostic context. The choice of diagnostic method is also affected by the purpose of the study. For some applications, such as declaration of freedom from the parasite, there is an urgent need to establish automated and ultrasensitive methods that are more precise, less time consuming and labor intensive.

The ability to diagnose stages of parasitic worms that are similar, and thus difficult to distinguish through morphological examination, has increased significantly with the emergence of various molecular technologies and, in particular, since the advent of polymerase chain reaction (PCR) methods based on examination of parasite DNA (8). The literature contains a number of examples describing how eggs and/or larvae of GIN, especially in sheep, can be determined to species level using different molecular diagnostic tools [for reviews, see Ref. (9, 10)]. Besides having the potential to be more sensitive than microscopy, molecular techniques can also be automated, and they are less time consuming than larval differentiation following fecal culture (11). The molecular tests, quantitative polymerase chain reaction (qPCR) and LAMP, would appear to have significant potential to improve our diagnostic capabilities in this area and help us understand whether nematode cross-transmission occurs between wild and domestic ruminants and whether it is a significant factor in development of resistance on livestock farms.

The aim of this study was to compare four diagnostic assays for detection of *H. contortus* eggs based either on microscopy or molecular methods. The scientific objective was to determine the presence of *H. contortus* by comparing results and evaluating the performance of each assay relative to the gold standard of conventional microscopy. This was considered essential to check the reliability and test performance of egg count data on *H. contortus* produced by a veterinary diagnostic laboratory in Sweden.

## Materials and Methods

### Parasite Material

Sheep feces were collected at a commercial laboratory in Sweden receiving ≈6,000 diagnostic samples on an annual basis. In total, 38 egg-positive samples originating from 17 naturally infected flocks were monitored for strongylid parasites. Individual samples of fresh feces were analyzed within 48 h of sampling by four different methods, of which two were based on detection of parasite eggs by microscopic examination and the remaining two by analysis with two different molecular diagnostic tools. All 38 samples contained strongylid eggs but were selected on the basis of both declared absence or presence of *H. contortus* and their individual egg counts after McMaster examination, as described below. The underlying intention was to have access to four sample groups: one without *H. contortus* (Negative group) and three additional groups with the parasite plus low, medium, and high egg counts.

### Animal Ethics

Feces samples were collected by licensed veterinarians from the rectum of sheep between June and July 2014, as part of routine diagnostic screening, and no ethical permission was required under Swedish legislation (Animal Welfare Act 2009/021).

### Microscopic Examination

Conventional egg counting was first performed with the McMaster method and then, after flotation and staining of eggs with peanut agglutinin (PNA), essentially as described by Jurasek et al. (12). McMaster egg counting was based on detection of nematode eggs in 3 g feces dispersed in 42 mL saturated NaCl, providing a diagnostic sensitivity of 50 eggs per gram feces (epg). Presence of *H. contortus* eggs were determined based on their characteristic shape, dark brown blastomeres, and body dimensions (average length = 70 ± 10 μm and width; with = 45 ± 5 μm). For PNA staining, eggs in 3 g feces were enriched by flotation with saturated NaCl, pipetted into new tubes, subjected to repeat centrifugation at 300 *g* for 5 min and washed several times in phosphate-buffered saline (PBS). In the staining process, the final pellet of parasite eggs was resuspended in 1 mL of PNA-FITC (Sigma Cat. No. L-7381 lectin from *Arachis hypogaea*, reconstituted at 5 mg/L mL PBS) and then incubated in dissolved lectin suspension for ≈1 h at room temperature. Before microscopic examination, egg samples were washed twice with PBS as described earlier and then transferred to a glass slide with 3 mL fluorescent mounting fluid (Fluoromount™ Aqueous Mounting Medium, Sigma F4680) and covered with a coverslip. Nematode eggs were viewed in the dark and lectin-binding intensity was recorded with an Olympus BH2-RFC fluorescence microscope. Three slides were produced for each sample, from which eggs were counted both under bright field illumination and when viewed with fluorescence.

### DNA Detection

Detection of *Haemonchus* DNA was performed following extraction and amplification either by real-time qPCR or by loop-mediated isothermal amplification (LAMP). The DNA extraction step of the total amount of eggs in 3 g feces was based on the Nucleospin Tissue Kit (Macherey-Nagel). In brief, flotated eggs were washed and transferred into Eppendorf tubes and incubated overnight at 56°C with proteinase K in lysis buffer (0.20 mg/mL) while being subjected to gentle shaking on a PCMT thermo-shaker (Grant-bio). DNA was then extracted according to the manufacturer’s instructions. Aliquots of the same DNA were examined with both methods, as described below.

In the qPCR analysis, species-specific *Haemonchus* primers targeting the internal transcribed spacer region (ITS2) of the ribosomal RNA gene array were used. The primer sequences employed were Hc forward 5′ GTT ACA ATT TCA TAA CAT CAC GT 3′ and Hc reverse 5′ TTT ACA GTT TGC AGA ACT TA 3′ (13). The qPCR reactions were carried out in a total volume of 25 µL reactions with QuantiTect SYBR Green PCR Kit (Qiagen) Kit: i.e., 12.5 µL 2× QuantiTect SYBR Green PCR Master Mix (Qiagen) and 0.3 µM of F and R primer, 2 µL DNA template and 10.5 µL molecular-grade water (2). The PCR cycle threshold (Ct) for all samples and controls, i.e., both no template control and positive control (DNA from adult *H. contortus*), was determined for identical technical duplicates in a Rotor-Gene 3000 (Corbett). Cycling conditions were: 95°C for 15 min followed by 45 cycles of 94°C for 15 s, 50°C for 30 s, and 72°C for 30 s. The results were analyzed using Rotor-Gene 6.1.90 software (Corbett).

The LAMP assay was carried out “blind” using prototype pelleted LAMP reagents (V6.21) designed by MAST Group Ltd. (Liverpool, UK), adapting the assay described by Melville et al. (14). In brief, LAMP mastermix was prepared as per the manufacturers’ protocol; four reagent pellets were resuspended in 344 µL 0.1 M Tris, 16 µL primer mix was added [1.6 µM FIP/BIP, 0.8 µM FLP/BLP, and 0.2 µM F3/B3, primer sequences as described by Melville et al. (14)], and 1 µL of template DNA was added to each reaction to give a final volume of 10 µL. Primer sequences; the LAMP reaction was incubated in a real-time PCR machine (ABI 7500) using the following conditions to keep a constant temperature and record fluorescence every 33 s; 60 cycles of 61°C for 32 s, 60°C for 1 s, giving a total reaction time of 33 min. Fluorescence was recorded using the FAM filter, and results were analyzed using the ABI 7500 software.

### Statistical Analysis

Graphs were prepared and differences between groups were tested with the Kruskal–Wallis non-parametric test followed by Dunn’s multiple comparison in GraphPad Prism6. This software was also used to compare test performance with Bland–Altman plots and Spearman rank correlation. All statistical tests were considered significant at *p* < 0.05.

## Results

### Microscopic Examination

**Figure 1:**
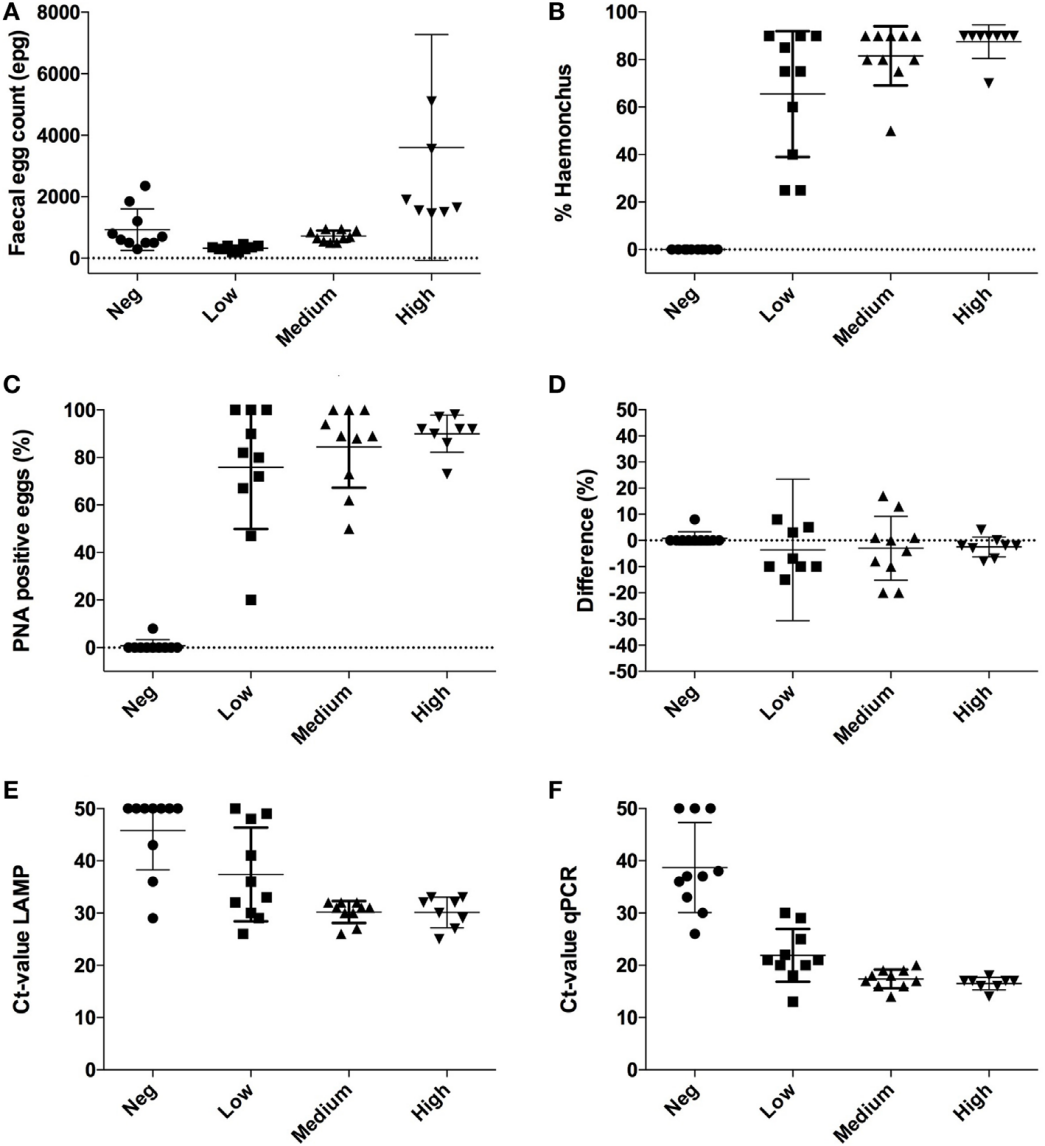
Individual results obtained with four different diagnostic methods applied to subsamples of fresh feces from 38 sheep. Samples were divided into four groups with samples from 8 to 10 sheep based on their nematode fecal egg count (FEC) and presence or absence of *H. contortus* following McMaster examination. **(A)** FEC expressed as eggs per gram feces (epg). Note one extreme value in the group “High” with 12,100 epg is outside the graph; **(B)** proportion of eggs identified as *H. contortus* in the McMaster chamber, **(C)** proportions of eggs in the samples that were stained with peanut agglutinin (PNA); **(D)** comparison between *H. contortus*-positive eggs with McMaster and following the PNA test; **(E)** cycle threshold (Ct) values with real-time quantitative polymerase chain reaction (qPCR); **(F)** Ct values with loop-mediated isothermal amplification (LAMP). For graphical reasons, negative results in both molecular tests are replaced with a Ct value of 50.

**Figure 2:**
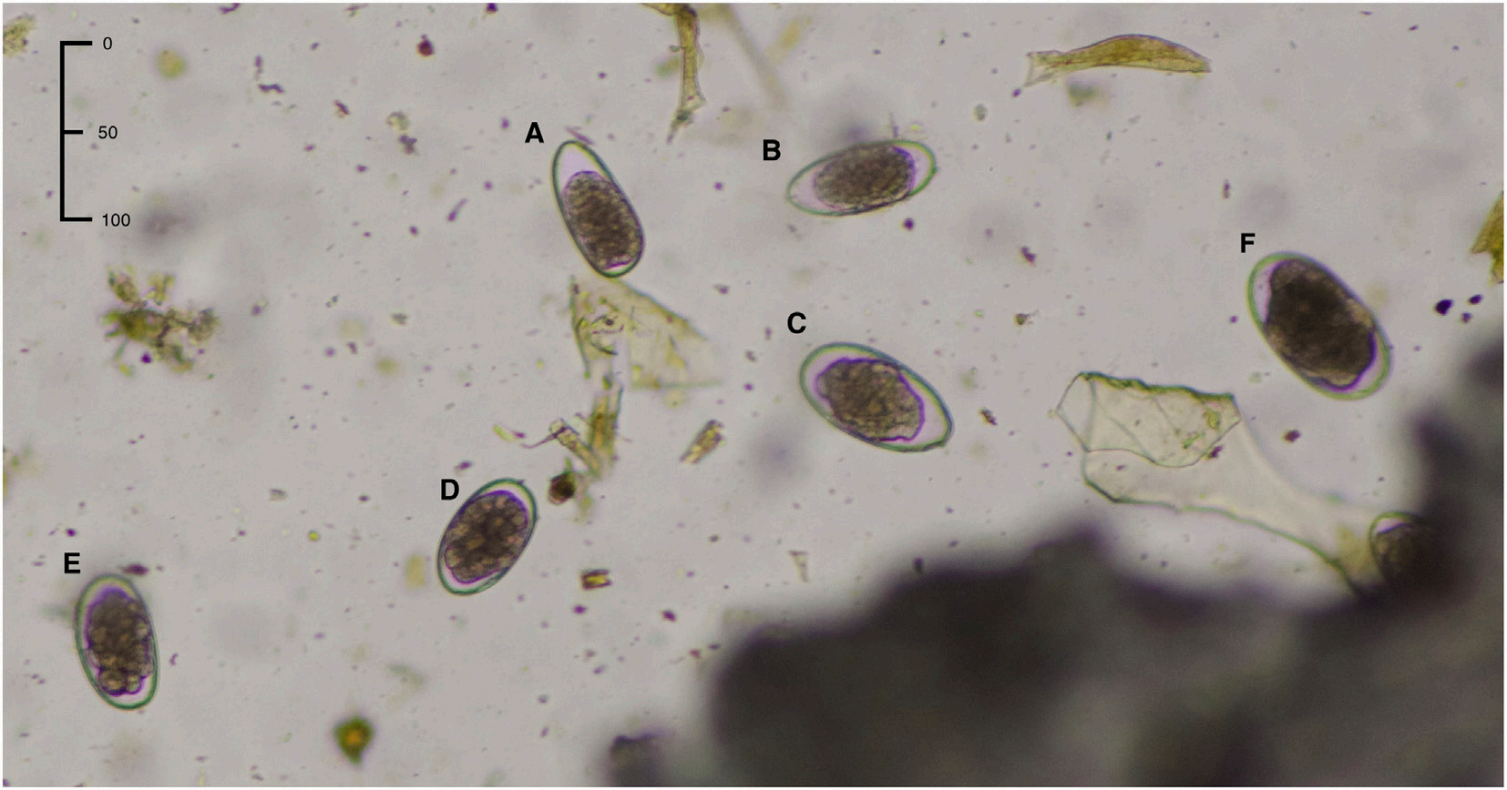
Morphological appearance of the strongylid eggs: **(A,B)** *Trichostrongylus axei*, **(C)** *Teladorsagia circumcincta*, **(D)** *Haemonchus contortus*, **(E)** doubtful egg type (cannot be classified as *Haemonchus*), **(F)** *Chabertia*/*Oesophagostomum*. Characteristics of *H. contortus* are length 70 ± 10 μm and width 45 ± 5 μm plus well-developed distinct blastomeres.

McMaster fecal egg counts (FECs) showed that all samples tested (*n* = 38) contained strongylid eggs, in numbers ranging from 200 to 12,100 epg (mean ± SD = 1,278 ± 2,049) (Figure 1A). According to morphological appearance (Figure 2), eggs of *H. contortus* were not present in 10 samples with a mean strongylid epg of 930 ± 672 (Negative group). The remaining 28 *H. contortus*-containing samples were divided into three groups based on their FEC level: Low (*n* = 10, range 200–499 epg), Medium (*n* = 10, 500–999 epg), and High (*n* = 8, ≥1,000 epg). The mean epg ± SD in these groups was: 328 ± 82 in Low, 720 ± 180 in Medium, and 3,600 ± 3,673 in High, and the proportion of *H. contortus* eggs was 69 ± 34, 85 ± 17, and 90 ± 8%, respectively (Figure 1B). Both the egg counts and the proportion of eggs classified as *H. contortus* differed significantly (*p* < 0.0001) between the groups.

These results were basically confirmed by lectin staining, which showed that only two out of 209 eggs (1%) were PNA-positive in one of the 10 samples deemed to be *H. contortus-*negative following McMaster examination. By contrast, lectin-stained eggs were found in all samples in the three groups (Low, Medium, and High) identified as being *H. contortus*-positive. A total of 79% (73/92), 85% (355/417), and 91% (486/535) of the eggs in the Low, Medium, and High groups tested PNA-positive when examined with fluorescence (differences not significant, *p* = 0.59) (Figure 1C).

**Figure 3:**
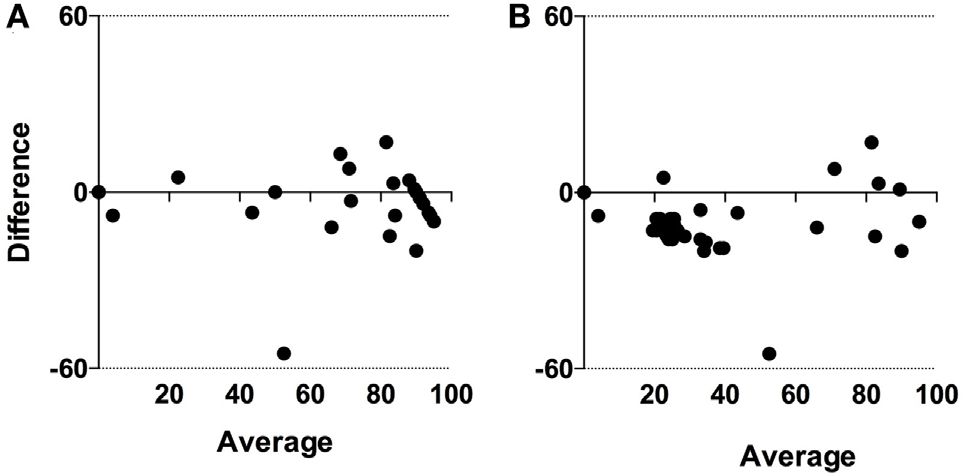
Bland–Altman limits of agreements between diagnostic tests based on **(A)** microscopy and **(B)** molecular detection. For graphical reasons, negative results with both molecular tests are replaced with a cycle threshold (Ct) value of 50.

Differences in the proportion of eggs identified as *H. contortus* by McMaster and after lectin staining with PNA are shown in Figure 1D. In general, there was good agreement except for one sample in the Low group. The agreement between McMaster and PNA results was confirmed by Bland–Altman plots (bias = −4.2 ± 11.4, 95% limit of agreement from −26.6 to 18.1) (Figure 3A).

### Molecular Results

In the LAMP analysis, seven samples (19%) had isothermal (Ct) values >35, and eight (22%) samples had no reaction. In the qPCR analysis, three (8%) samples had a cycle threshold (Ct) >35, and only three (8%) showed no reaction. Those samples where no increase was observed were replaced by a value of 50 for graphical reasons. With LAMP, the equivalent of Ct values ranged between 25 and 50 and differed significantly (*p* = 0.0011) between the sample groups. The value was 46 ± 7.5 (mean ± SD) for samples in the Negative group, 37 ± 9.0 in Low, 30 ± 2.0 in Medium, and 30 ± 2.9 in High (Figure 1E). The Ct values obtained with qPCR ranged between 13 and 38 and also differed significantly (*p* < 0.001) between the sample groups. The value was 34 ± 4.6 (mean ± SD) in the Negative group, 22 ± 5.0 in Low, 17 ± 1.8 in Medium, and 17 ± 1.2 in High (Figure 1F).

**Figure 4:**
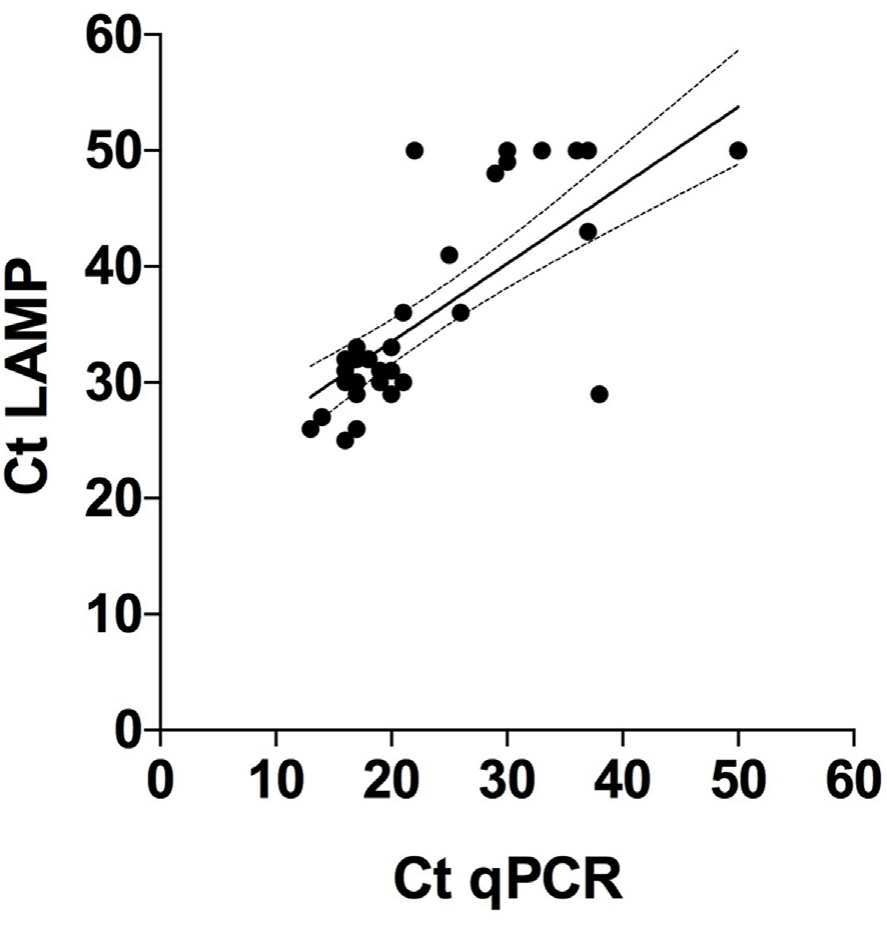
Correlation between cycle threshold (Ct) values obtained with quantitative polymerase chain reaction (qPCR) and loop-mediated isothermal amplification (LAMP) on DNA extractions from 38 individual sheep feces samples tested with these diagnostic methods. Tests that gave no results were arbitrarily set to 50. Spearman rank = 0.7 (*p* < 0.0001), 95% confidence interval = 0.6 and 0.9 for qPCR and LAMP, respectively.

When comparing Ct values in Figure 1E (LAMP) and Figure 1F (qPCR) it is obvious that the Ct values generated with qPCR are overall lower than with LAMP. However, Spearman rank correlation between the two sets of test results was highly significant (*p* < 0.001; *r* = 0.7; 95% CL 0.6–0.9) (Figure 4). The agreement between these tests was further confirmed in the Bland–Altman plot (bias = −9.8 ± 10.1, limit of agreement from −29.6 to 10.0) (Figure 3B).

## Discussion

In this study, there was generally good agreement between the four different diagnostic methods compared. The two molecular methods were more sensitive than the two based on microscopy, but the discrepancy between the microscopy methods (−4.2 ± 11) was less than for the molecular tests (−9.8 ± 10). However, both molecular diagnostic tests, and especially qPCR, were more sensitive in detection of *H. contortus* than conventional microscopy.

Strongylid eggs look very similar, but small differences in egg shape, color, and morphology can still be used for identification of different species, although there is some overlap between different genera and/or species infecting sheep (6). However, correct identification of nematode eggs, especially the multitude of species present in feces samples, requires highly skilled expertise, which is not always readily available. It has also been claimed that morphological examination is not feasible in a routine diagnostic context, because the eggs must be viewed and measured at high magnification (9). Thus, over the years lectin-binding assays with PNA, which stains specifically for *H. contortus* eggs, have been developed, further validated and refined (12, 15, 16). Despite this development work, to the best of our knowledge, the fluorescein-labeled PNA test has not been widely adopted in diagnostic laboratories, possibly because examination requires access to an advanced microscope equipped with fluorescent light. Besides, we have found that lectin staining with PNA is tedious and time consuming to perform. Thus, this test is not feasible when it is necessary to examine a large number of eggs per feces sample. Another limitation with PNA is that it is difficult to quantify. Taken together, this makes the PNA test cumbersome, especially in routine diagnostic settings.

According to our results, only 2 out of more than 200 eggs was PNA-positive in the group deemed to be *H. contortus*-negative following McMaster counting and, moreover, both of these eggs were observed in the same sample. This indicates that most eggs were correctly identified in the Negative group without further processing, and thus eggs of this parasite are obviously not overdiagnosed. However, it also indicates that there is a certain risk of *H. contortus* being missed when ovine fecal samples are examined by McMaster only. This implies that fecal diagnosis should be refined, especially in cases when it is of particular importance to identify the eggs of *H. contortus* with high accuracy. This may arise in connection with examination of feces after quarantine treatment with anthelmintics, for example, in relation to movements of wild or domestic animals between countries. The gain from staining the eggs with PNA in this context seems to be minimal, especially when the feces samples examined are from several animals in the same flock, when it is vitally important to find out if the parasite is present or absent.

Interestingly, the values indicating presence of *Haemonchus* DNA were observed both with qPCR and LAMP for the single sample from the *H. contortus* “Negative” group that contained PNA-positive eggs. This in itself further illustrates that PNA staining is superior and more sensitive than conventional McMaster counting.

On comparing the outcomes of the molecular testing, it was evident that more samples examined by qPCR (*n* = 34) were positive compared with samples examined by LAMP (*n* = 29). This illustrates that qPCR is superior to LAMP, although it is an open question whether the Ct values are directly comparable between these tests. Another study on *H. contortus* eggs isolated from ovine samples has reported 10-fold higher sensitivity of results obtained by fluorescent LAMP compared with conventional PCR (14). Although we used the same LAMP primer set in this study, the reagents used contained a different chemistry allowing for translation to a real-time PCR platform. The Ct results obtained with qPCR in our study were also based on a slightly different protocol for ITS2 detection (13) than in the conventional PCR used by Melville et al. (14). Although there is no doubt that both tests generated congruent results (Spearman *r* = 0.7), it is difficult to make direct comparisons between Ct values obtained with these molecular methods, as the thresholds obviously differ between the methods (Figure 1). It is also evident that more samples reacted when they were tested with qPCR compared with LAMP, both in the *H. contortus*-negative and Low groups. Furthermore, the Ct values obtained with qPCR were in general lower than those obtained with LAMP.

It was beyond the scope of this study to investigate whether it is practical to set up and offer these techniques in a smaller diagnostic setting receiving routine samples from veterinarians. Still, LAMP in particular seems to provide a viable option, even in a small diagnostic laboratory, because it can generate highly sensitive and reliable results in less than an hour even in a resource-limited situation. By contrast, qPCR requires more sophisticated equipment, such as a real-time PCR thermal cycler.

From this study, it can be concluded that in most situations it is possible to identify *H. contortus* in sheep with the McMaster method at flock level, without making major mistakes with false positives. However, both PNA and especially the two molecular methods were obviously more sensitive than microscopy. Although this increased sensitivity is not always required for high-throughput diagnostic laboratories, it is vital for certain purposes, for example, when it important to avoid the spread of isolates of *H. contortus*, especially if they are resistant to anthelmintics.

## Author Contributions

Conceptualization and funding acquisition: JH. Acquisition of data: SL and LM. Analysis and interpretation of data and drafting of manuscript: JH and PS. Critical revision: all the authors.

## Conflict of Interest Statement

The authors declare that the research was conducted in the absence of any commercial or financial relationships that could be construed as a potential conflict of interest. The reviewer BP and handling editor declared their shared affiliation.
